# VA-ECMO and HIET for toxic cardiogenic shock in an elderly patient following severe poly-intoxication: A case report

**DOI:** 10.1016/j.toxrep.2026.102307

**Published:** 2026-07-04

**Authors:** J.J.J. Ouwerkerk, N.A. Poublon, A.F. Westra, N. Kraaijvanger

**Affiliations:** a Emergency Department, Leiden University Medical Centre, Leiden, the Netherlands; b Intensive Care Department, Leiden University Medical Centre, Leiden, the Netherlands

**Keywords:** ECMO, Intoxication: HIET, Tricyclic antidepressants, Calcium channel blocker, Beta blocker

## Abstract

This case report presents the successful use of high-dose insulin euglycemic therapy (HIET) and venous arterial extracorporeal membrane oxygenation (VA-ECMO) in managing severe toxic cardiogenic shock following poly-intoxication in an elderly patient. A 67-year-old woman, with a history of depression, previous suicide attempts and chronic renal failure, overdosed on multiple cardiovascular active agents including metoprolol (β-blocker), nifedipine (calcium channel blocker), nortriptyline (tricyclic antidepressant), venlafaxine (serotonin–noradrenaline reuptake inhibitor), and temazepam (benzodiazepine) were reported. Initial treatment with vasopressors, HIET and supportive measures stabilized her condition temporarily. Despite these interventions, the patient developed catecholamine-refractory shock with progressive multiorgan failure, requiring escalation to VA-ECMO as a bridge to recovery. VA-ECMO resulted in hemodynamic stabilization, enabling vasopressor withdrawal and decannulation after three days. The patient survived the acute toxic phase without irreversible neurological injury. However, the course was complicated by vascular complications, prolonged mechanical ventilation, renal replacement therapy, severe functional decline and eventually death eight months later. This case illustrates that VA-ECMO may provide effective temporary circulatory support in severe reversible toxic cardiogenic shock, even in elderly patients with significant comorbidity. However, the substantial burden of long-term morbidity and prolonged recovery should be carefully weighed during multidisciplinary decision-making.

## Highlights

• TCA, CCB, BB and SNRI overdoses remain among the most lethal drug intoxications, resulting in refractory shock.
• HIET can provide temporary hemodynamic stabilization in severe cardiotoxic poly-intoxication; however, it should be initiated early for maximal effect.
• Advanced age and comorbidity should not automatically preclude VA-ECMO in reversible toxic shock.
• Long-term functional outcome, frailty, and rehabilitation burden should be considered in elderly VA-ECMO candidates.

## 1 Introduction

Medication overdoses are a substantial public health burden, accounting for an estimated 0.5–1% of all emergency department (ED) visits in high-income countries, of which up to 70% are intentional and related to self-harm or suicidal behaviour. [1] According to the 2023 America’s Poison Centers Annual Report, calcium channel blockers (CCB) and beta-blockers (BB) rank among the most frequent causes of fatal drug exposures (ranked #6 and #7, respectively). [2] Other psychotropic agents, including serotonin–noradrenaline reuptake inhibitors (SNRI) and tricyclic antidepressants (TCA), also account for fatal exposures, albeit less commonly (#21 and #23). [2] The lethality from CCB and BB is the result of a combination of profound cardiogenic and vasodilatory shock; [3], [4] Myocardial depression from inhibition of cardiac membrane channels and receptors reduces contractility, together with severe vasoplegia, produces severe catecholamine-refractory hypotension. [3], [4] This risk is further increased in cases of combined intoxication involving multiple classes of cardiovascular active agents. [3], [4].

The 2023 American Heart Association (AHA) guideline recommends managing cardiogenic shock from an overdose of these cardiovascular active agents by reversing the underlying toxicity while providing circulatory support. [5] Although vasopressors/inotropic agents can help maintain organ perfusion, their efficacy may be limited in toxin-induced shock. [6] Therefore, effective treatment often requires specific or advanced therapies, including calcium, glucagon, high-dose insulin euglycemic therapy (HIET), and venous arterial (VA) extracorporeal membrane oxygenation (ECMO) as rescue support in refractory cases. [5], [7].

ECMO is commonly used in severe respiratory and cardiovascular compromise such as cardiogenic shock, acute respiratory distress syndrome (ARDS) and some forms of refractory cardiac arrest. [8] Whilst not being a definitive treatment for the underlying pathologies, it’s often employed as a rescue therapy and utilised as a bridge to recovery, a bridge to transplantation or occasionally a bridge to decision. Over the last years, also its role as bridge to recovery in toxic cardiogenic shock has gained increasing recognition. [5], [7], [8].

This case highlights the potential role of VA-ECMO in managing severe toxic cardiogenic shock following poly-intoxication in an elderly patient. It aims to raise awareness among clinicians that these advanced supportive therapies can be considered when conventional treatment fails, even in the elderly with comorbidities, while acknowledging both their potential benefits and associated complications.

## 2 Case report

A 67-year-old female patient was found by emergency medical services (EMS) smoking a cigarette outside her apartment approximately 30 min after a suicide attempt involving the ingestion of multiple medications, including but not limited to: metoprolol, nifedipine, nortriptyline, venlafaxine, and temazepam, all at toxic doses (a comprehensive list of the reported medications, their dosages, and mechanisms of action can be found in Table 1). After ingestion, the patient contacted psychiatric services to convey a farewell message, leading to EMS being alerted. Her medical history was notable for depression, iron deficiency anaemia, hypertension, reduced renal function attributed to previous lithium use, and prior suicide attempts. Her estimated bodyweight was 90 kg. At EMS arrival, she was hemodynamically stable with normal vital signs.

**Table 1:** Characteristics and dosages of ingested drugs.

Drug	Estimated ingested dose	Maximum recommended therapeutic daily dose*	Substance Class	Mode of Action	Symptoms of overdose
Metoprolol, prolonged-release [9]	14 × 100 mg (1.400 mg)	200 mg [10]	Selective β-blocker	β₁-adrenergic receptor blockade	Bradycardia, hypotension, decreased cardiac function
Nifedipine, prolonged release [11]	10 × 30 mg (300 mg)	120 mg [12]	dihydropyridine calcium channel blocker	L-type calcium channel blockade	Hypotension, decreased cardiac function
Nortriptyline [13]	42 × 25 mg (1.050 mg)	150 mg [14]	Tricyclic antidepressant	Sodium channel blockade, α-adrenergic receptor antagonism, and inhibition of norepinephrine/serotonin reuptake	Dysrhythmia, CNS depression or stimulation, hypotension seizures, anticholinergic syndrome, decreased cardiac function
Venlafaxine [15]	60 × 75 mg (4.500 mg)	375 mg [16]	Serotonin–noradrenaline reuptake inhibitors	Blockade of neuronal serotonin and norepinephrine reuptake	Seizures, dysrhythmia, serotonin syndrome
Temazepam [17]	11 × 20 mg (220 mg)	40 mg [18]	Benzodiazepine	GABA-A receptor potentiation	CNS depression, respiratory depression, coma, decreased cardiac function
Pravastatin [19]	14 × 20 mg (280 mg)	40 mg [20]	Statin	HMG-CoA reductase inhibition	Gastrointestinal discomfort, myalgia, hepatic dysfunction (rare)
Sodium bicarbonate (oral) [21]	38 × 500 mg (19.000 mg)	2000 mg [21]	Alkalizing agent (antacid)	Alkalizing agent	Gastrointestinal discomfort

Dosages were estimated by the emergency medical team (EMS) based on the empty medication strips and containers found at the scene; EMS provided a detailed list of medications during handover. As these estimates were based on the medication packaging found at the scene, uncertainty regarding the exact ingested dosages remains. No serum drug levels were obtained, as the clinical presentation was considered highly consistent with severe intoxication and these were not expected to alter management decisions.

* Maximum recommended therapeutic daily doseswere retrieved from the Dutch Formulary.

cAMP = Cyclic adenosine monophosphate; GABA = Gamma-aminobutyric acid; HMG-CoA = β-Hydroxy β-methylglutaryl-CoA; LDL = low-density lipoprotein; HDL = high-density lipoprotein; CNS = CNS depression.

45 min after ingestion and during EMS transport, the patient deteriorated from full responsiveness with stable vitals to decreased mental status (E2M5V2). On reassessment patient was tachycardic at 115 bpm, with a blood pressure of 86/48 mmHg, a respiratory rate of 10 breaths/min and an oxygen saturation of 92% on room air.

The patient presented at the emergency department (ED) approximately one hour after ingesting the overdose. Her condition had already worsened significantly during transport and continued to deteriorate in the ED. Ten minutes after arrival, her manual blood pressure readings were nearly unmeasurable, occasionally recording 50/30 mmHg. Her heart rate declined to 65 beats per minute. Her consciousness dropped further to E1M1V1 at this stage. Despite administration of 15 L/min oxygen through a non-rebreather mask (NRBM) and the use of two nasopharyngeal airway devices, her oxygen saturation decreased from 92% to ultimately 40%. This was attributed to a combination of airway obstruction and decreased respiratory drive as a result of reduced level of consciousness, ventilation-perfusion mismatch and inadequate pulse oximetry readings due to profound hypotension.

Bag-mask ventilation was initiated and after she had been treated with two grams calcium gluconate intravenously, 5 mg glucagon, 100cc sodium bicarbonate 8.4% and 1500 mL of crystalloid fluids initially, vasopressors were started with a total of seven 50 µg boluses of phenylephrine and a norepinephrine infusion titrated up to 0.93 µg/kg/min. These interventions raised her blood pressure to 95/55 mmHg, as confirmed by invasive monitoring through an arterial line. With increasing cerebral and peripheral perfusion pressure, spontaneous ventilation returned, as well as pulse oximetry reading 92% and her consciousness improved to E1M5V2.

The first electrocardiogram (ECG) in the ED, performed 90 min after the overdose, showed a slightly prolonged QRS (109 ms). However, the ECGs later on in the intensive care unit (ICU) demonstrated an increasingly prolonged QRS (max 135 ms) with a prominent terminal R wave in aVR, suggesting sodium channel blockade. Further, with an absolute QT interval of 400 ms at an heart rate of 78 bpm, the QT nomogram indicated no increased risk of torsades de pointes. [22] At the ED, her venous blood gas demonstrated a pH of 7.30, with pCO^2^ of 7.9 kPa, lactate of 2.4 mmol/L and a glucose of 5.6 mmol/L. All other laboratory results were unremarkable except for her known renal failure, indicated by an eGFR of 17 mL/min/1.73 m². Approximately 3 h after ingestion she was transferred to the ICU for further monitoring and treatment. Activated charcoal was considered; however, this was deferred because of rapidly progressive hemodynamic instability, reduced consciousness, and the absence of a definitively secured airway.

**Fig. 1:**
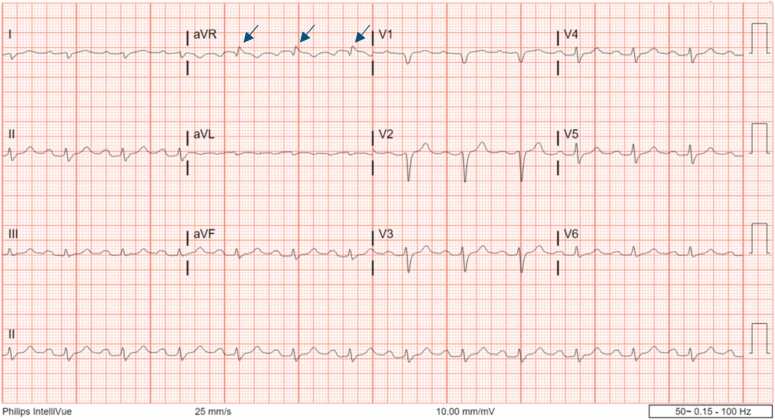
ECG 4 h post-ingestion in ICU. The ECG demonstrates sinus rhythm at 78 bpm with right axis deviation. A prolonged QRS duration of 135 ms is present, while the QTc interval is mildly prolonged at 467 ms. Additionally, a prominent terminal R wave in lead aVR (blue arrow) is observed, consistent with sodium channel blockade seen in TCA intoxication.

Upon arrival in the ICU her consciousness remained decreased at E1M5V2. Patient was breathing spontaneously and her oxygen saturation maintained above 90% on 15 L/min oxygen through a NRBM. Given the patient’s hemodynamic instability and ongoing requirement for high-dose vasopressor support, endotracheal intubation was deferred at this stage. The priority was initial hemodynamic resuscitation, as induction of anesthesia with additional hypnotic drugs was considered to carry a high risk of precipitating further cardiovascular collapse. A second generation supraglottic airway device (SAD) was therefore placed, which she was able to tolerate well without the need for additional sedatives. Spontaneous ventilation and oxygenation kept being adequate with low inspiratory pressures, minimal leakage and FiO2 was titrated down to 0.5. The patient hemodynamically stabilised and norepinephrine infusion was at that point decreased to 0.27 µg/kg/min.

Eight hours post-ingestion, there was a secondary decline in the patient’s condition. The level of consciousness decreased to E1M1V1. The patient was then intubated to reduce the risk of aspiration of gastric contents owing to decreased airway reflexes. Concurrently there was a marked decrease in diuresis and a lactate increase on arterial blood gas to a maximum of 4.7 mmol/L and ventilator FiO2 needed to be increased to 0.8 after intubation. This was attributed to acute progression of left sided cardiac failure, which was confirmed by severely decreased left ventricular function on echocardiography and chest x-ray indicating pulmonary congestion.

At 11 h post-ingestion, high-dose insulin euglycemic therapy (HIET) was initiated because of the progression of the toxic cardiogenic shock. Treatment consisted of an initial bolus of 80 units of insulin with 40 g of glucose, followed by a continuous infusion of insulin at 80 units/hour with concurrent glucose at 40 g/hour following the relevant guideline of the Dutch National Poisoning Information Center. [23] Concomitant glucose supplementation was titrated to maintain euglycemia, with frequent glucose and electrolyte monitoring during treatment. Particular attention was paid to potassium levels because HIET may induce hypokalemia. The intended therapeutic response was improvement in perfusion parameters, including blood pressure, lactate clearance, urine output, and vasopressor requirement. Additionally, inotropic support with dobutamine at 5 µg/kg/min and milrinone at 0.25 µg/kg/min was initiated, while the continuous adrenaline infusion was maintained.

Despite these measures, blood pressure dropped significantly to 78/55 (MAP 57) and serum lactate remained elevated and diuresis nearly stopped. Norepinephrine infusion was increased to 0.59 µg/kg/min and argipressin at 0.01IE/min was started to treat profound vasodilation, with minimal effect. Additionally, the patient was treated with 3 g of calcium gluconate, 5 mg glucagon, 1000 mL of crystalloid fluids (totalling 2500 mL), and 100 mL of 8.4% sodium bicarbonate, administered in response to QRS rewidening (which increased to a maximum of 145 ms in ICU). At that stage, the patient was considered to have developed catecholamine-refractory shock, based on persistent hypotension with MAP < 65 mmHg, rising lactate levels indicating ongoing tissue hypoperfusion, near-anuric renal failure, worsening respiratory failure, and severely depressed left ventricular function on echocardiography, despite maximal conventional therapy including escalating vasopressor and inotropic support as described above and HIET; Given the patient's refractory cardiogenic shock, VA-ECMO as a bridge to recovery was considered. In our local ICU-protocol regarding VA-ECMO, refractory cardiogenic shock is defined as persistent systolic blood pressure < 90 mmHg with a cardiac index < 2.2 L/min/m² and/or a cardiac power output < 0.6 W despite treatment with two or more vasoactive agents. In the absence of other contraindications (advanced age over 70 years, severe aortic regurgitation, aortic dissection, cardiac arrest with uncertain neurological outcome, a contraindication for systemic anticoagulants such as recent intracranial haemorrhage or metastasised malignancy), a consultation with psychiatry was held prior to cannulation, to consider the appropriateness of this treatment modality given the suicide attempt and the patient's medical and psychiatric history. Considering the patient's positive response to treatment of a previous episode of depression, the relapse leading to this intoxication was deemed reversible and sixteen hours after ingestion VA-ECMO was initiated. ECMO placement was percutaneously. The left femoral vein was canulated with a 25 French drainage canula and the right femoral artery was canulated with a 19** French return canula. A distal perfusion canula was placed in the right superficial femoral artery which is common practice with this VA-ECMO configuration in our institution. Invasive blood pressure was already monitored in the right radial artery and INVOS (Medtronic plc, Minneapolis, USA) monitoring was applied bilaterally on the forehead and distally on the lower limbs to possibly detect differential hypoxemia and/or a relative perfusion defect to the right lower leg. Initial hemodynamic goals in our institution are ECMO flow of 3–4.5 L/min in adults, further titrated on a target MAP> 65 mmHg, SvO2 of > 60% and a normalisation of the serum lactate, while sweep flow is titrated to achieve an arterial pH between 7.35 and 7.45. Transthoracic echocardiography is conducted daily to evaluate opening of the aortic valve and unloading/distension of the left ventricle (LV), LV function, mitral valve regurgitation, right ventricular function and pericardial effusion. Directly after connecting the ECMO circuit a bolus of 5000IE iv heparin is administered followed by a continuous infusion with 6-hourly dose titration targeted on anti Xa levels between 0.15 and 0.30 IU/mL. Initial ECMO flow was 2.7 L/min. Lactate decreased to normal levels, norepinephrine could be tapered to a minimal dose and diuresis increased slightly over the subsequent hours. Directly after cannulation, a pale and slightly colder left foot and no duplex signal distal of the femoral artery were noticed. A vascular surgeon was consulted and additional imaging studies were conducted. Thrombotic material in the external iliac artery and superficial femoral artery and an occlusion of the popliteal artery were detected, which were all effectively managed with additional intravenous heparin. In the subsequent days, vasopressor support was gradually discontinued, and transthoracic echocardiography showed recovery of the systolic function of the left ventricle and no other abnormalities. In our hospital, patients are eligible for weaning of VA-ECMO as soon as the underlying cause of cardiogenic shock has subsided, pulse pressure has increased to > 20 mm Hg for over 24 h and the earlier mentioned hemodynamic goals are met with decreasing dose of supportive inotropes (dobutamine <5 mcg/kg/h) and vasopressors (norepinephrine <0.1 mcg/kg/h). ECMO flow is then initially tapered down to 2,5 L/min and a wean trial is conducted when anti-Xa levels are 0.5IU/mL. In our institution ECMO-flow is decreased to 0.5 L/min for 15 min during this wean trial. When pre-defined hemodynamic (CVD<15 mmHg, MAP >65 mmHg, pulse pressure >30 mmHg, ScvO2 >60%, lactate <2 mmol/L, VIS score <15) and echocardiographic (LVOT VTI ≥12 cm, TSDa ≥6 cm/sec and LVEF ≥20–25%) criteria are met, the patient is eligible for VA-ECMO removal. The patient met these criteria after three days and was then successfully weaned from VA-ECMO.

During decannulation, which in our hospital is conducted in the ICU after initial percutaneous ECMO placement and canula insertion openings are routinely closed with purse-string sutures, she lost 800 cc of blood from her right common femoral artery, necessitating a secondary transport to the OR for embolectomy and surgical closure of the right femoral artery. An additional embolectomy was later performed to resolve a right popliteal artery occlusion, likely due to embolic material migrating distally during the femoral artery procedure.

Her ICU stay was further complicated by respiratory insufficiency due to ventilation associated pneumonia and a reintubation due to ICU acquired weakness (ICUAW) and atelectasis, necessitating a tracheostomy to wean her from the mechanical ventilation. Multifactorial acute-on-chronic renal insufficiency due to the hemodynamic collapse, bleeding and intravenous contrast exposure was managed with continuous venovenous hemofiltration until renal function returned to its baseline level. After 22 days in the ICU, she was transferred to the hospital's psychiatric ward primarily for observation of ICUAW. After a total of 66 days, she was discharged from the hospital to a rehabilitation centre. At hospital discharge, no ongoing depressive symptoms or suicidal ideation were present, and the patient expressed regret regarding the suicide attempt together with a wish to continue living. Neurological examination showed no focal deficits, although persistent bilateral foot drop and ICU-acquired weakness which required rehabilitation. Renal function partially recovered, with an estimated glomerular filtration rate (eGFR) of 28 mL/min/1.73 m² at discharge. See Fig. 2 for an overview for the major stages of clinical deterioration and escalation of therapy.

**Fig. 2:**
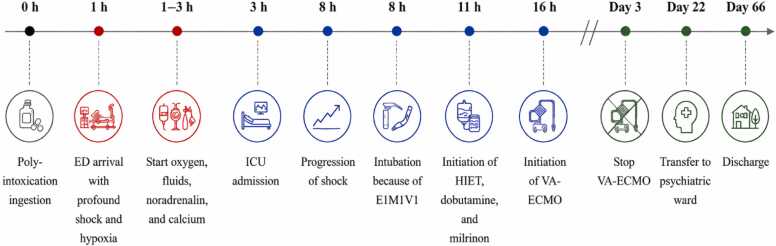
Timeline of clinical deterioration and escalation of therapy. ED = emergency department; ICU = intensive care unit; HIET = high-dose insulin euglycemic therapy; VA-ECMO = venous arterial extracorporeal membrane oxygenation.

The subsequent course remained complicated by pressure ulcers and osteomyelitis on her left heel, likely related to prolonged immobilization and use of a lower-leg splint for foot drop, ultimately requiring below-knee amputation due to insufficient antibiotics response. Several months later, the patient was readmitted with urosepsis which was treated with antibiotics and fluids. However, due to frailty and functional decline, further care was transitioned to a palliative approach, after which the patient died in the rehabilitation facility in approximal 8 months after intoxication.

## 3 Discussion

This case report describes a patient with catecholamine-refractory toxic cardiogenic shock following ingestion of BB, CBB, TCA, SNRI, and benzodiazepines at toxic doses, in whom HIET and eventually VA-ECMO proved life-saving. Table 1 and Fig. 3 provide an extensive overview of the underlying pathophysiology and drug interactions.

**Fig. 3:**
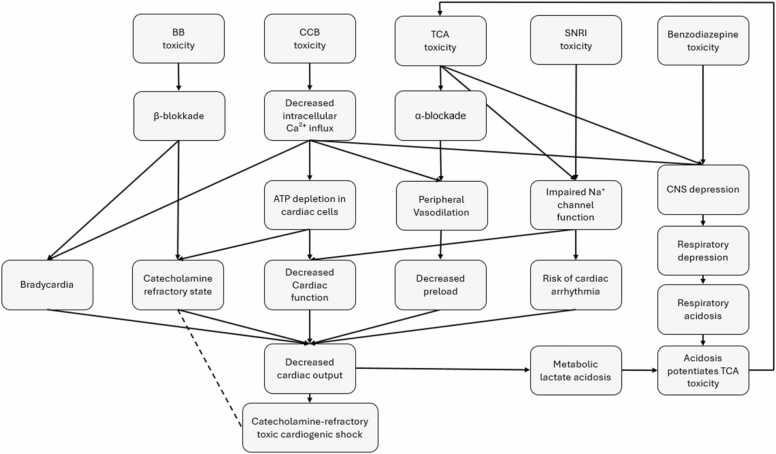
Overview of the pathophysiological mechanisms in the mixed drug toxicity of our patient. BB = Betablocker; CCB = Calcium Channel Blocker; TCA = Tricyclic Antidepressant; SNRI = Serotonin Noradrenaline Reuptake Inhibitor; CNS = Central Nervous system; ATP = Adenosine Triphosphate. Pravastatin and sodium bicarbonate were not included in the table, as their toxicity has minimal impact on haemodynamic collapse.

Metoprolol and nifedipine both reduce intracellular calcium influx through distinct mechanisms, resulting in decreased heart rate and vasodilation. [8], [9], [11] In BB toxicity, inhibition of β₁-adrenergic receptors in cardiac myocytes indirectly decreases calcium entry, reducing contractility and heart rate. [9] CCB toxicity, in contrast, directly blocks calcium influx through L-type channels across multiple tissues. [11] This means calcium-dependent depolarization in the sinoatrial and atrioventricular nodes is impaired, leading to slowed atrioventricular conduction and bradycardia. [11] Similarly, calcium channel blockade in the central nervous system impairs neuronal signal transmission, which may result in reduced levels of consciousness. [11] Additionally in CCB toxicity, the resulting calcium deficit leads to hypoinsulinaemia, as insulin release requires calcium influx into pancreatic β-cells, which reduces glucose availability to cardiac cell. [9], [11] This impairs myocardial metabolic adaptation by preventing the normal shift from fatty acid to glucose utilisation during stress. [9], [11] This occurs through reduced glucose uptake due to hypoinsulinaemia and inhibition of calcium-dependent mitochondrial oxidative metabolism, leading to decreased ATP production. [9], [11] All together, these mechanisms lead to catecholamine-refractory cardiogenic shock, where the myocardium is deprived of ATP, resulting in reduced cardiac output. [9], [11] BB toxicity further diminishes catecholamine responsiveness and causes bradycardia, while CCB toxicity adds peripheral vasodilation, creating a distributive component that further reduces preload and cardiac output. [9], [11].

Treatment of BB and CCB intoxication involves several complementary approaches. [5] Fluid resuscitation is recommended first. Intravenous calcium may help overcome intracellular calcium deficiency, improving contractility and vascular tone. [5] Ionotropic agents can be initiated, though their effectiveness may be limited because BB toxicity prevents catecholamines from stimulating the heart. [6] Glucagon bypasses the blockade by activating an alternative receptor pathway, which may restore heart rate and contractility through increased intracellular calcium levels, though studies show the response can vary. [5], [24] HIET is recommended as first-line treatment for BB and calcium CCB intoxication with cardiovascular compromise, as several studies and case reports have demonstrated favorable outcomes. [5], [24], [25] It enhances myocardial glucose use and ATP production, improving calcium handling and contractility. [5], [24], [26] As insulin-mediated inotropy is independent of β-receptor activity, it remains effective despite β-blockade. [5], [24], [26] HIET should be initiated early to interrupt the pathophysiological cascade of progressive myocardial dysfunction and circulatory shock. [23], [24] In this case, HIET was initiated approximately 11 h post-ingestion after a secondary clinical deterioration possibly related to delayed and erratic drug absorption from a pharmacobezoar,. However, his delay in HIET initiation may have contributed to the limited hemodynamic response. Moreover, current Dutch guidelines suggest escalation of HIET to insulin doses of up to 10 units/kg/hour. [23] Such dose escalation was not applied in this case, which may have further reduced the maximal therapeutic effect of HIET. Although current Dutch guidelines suggest escalation of HIET to insulin doses of up to 10 units/kg/hour in refractory cases, [23] further escalation was not pursued in this patient because of rapidly progressive circulatory collapse with evolving multiorgan failure despite ongoing therapy. At that stage, the treating team considered the likelihood of delayed benefit from additional HIET escalation to be outweighed by the immediate need for mechanical circulatory support with VA-ECMO.

Nortriptyline and venlafaxine inhibit multiple neurotransmitter uptake, increasing synaptic neurotransmitter levels. [13], [15] In TCA and SNRI toxicity, sodium channel blockade in cardiac myocytes impairs depolarization, decreases contractility, and increases arrhythmia risk, first seen as QRS prolongation, later as ventricular tachycardia or asystole. [13], [15] These electrical disturbances reduce contraction efficiency and cardiac output. [13], [15] At high doses, TCA toxicity also causes CNS depression through anticholinergic and antihistaminic effects, worsened by concurrent benzodiazepine use via GABA stimulation and CCB induced suppression of neuronal calcium-dependent synaptic transmission. [11], [13], [15], [17] The resulting CO₂ retention from hypoventilation, together with lactic acidosis caused by the impaired perfusion seen in CCB and BB intoxication in this case, drives progressive systemic acidosis. In this acidic state, sodium channel blockers, particularly TCAs, bind more strongly and dissociate more slowly, further impairing cardiac function, causing progressive QRS widening, and increasing arrhythmia risk. [13].

Severe TCA and SNRI toxicity require haemodynamic support with adequate/mechanic ventilation to maintain an adequate pH and vasopressors; [13], [15] however, in this case, vasopressor response was limited due to concurrent CCB and BB intoxication. Additionally, maintaining a pH of 7.45–7.55, achieved through sodium bicarbonate therapy or, if needed, controlled ventilation to induce mild hypocapnia, reduces TCA toxicity by decreasing sodium channel blockade and improving cardiac conduction. [13] Benzodiazepine toxicity can be treated with flumazenil, a specific antidote; [17] however, it is contraindicated in poly-intoxication because it may precipitate seizures or convulsions, particularly when co-ingested with TCAs or other pro-convulsant agents. [17].

According to the AHA and European Resuscitation Council, VA-ECMO is indicated in poisoning when patients continue to deteriorate despite maximal supportive care, often referred to as catecholamine-refractory shock. [5], [27] Importantly, catecholamine-refractory shock is not uniformly defined, but generally refers to persistent circulatory failure with ongoing tissue hypoperfusion despite escalating vasopressor and inotropic support and maximal conventional therapy. [28] In this context, VA-ECMO provides mechanical circulatory support to manage refractory cardiogenic shock while the offending toxin is cleared. Observational data suggest that patients, with refractory shock due to poisoning, who are placed on VA-ECMO have lower mortality than both non-poisoned VA-ECMO patients and poisoned patients treated with standard critical care and antidotal therapy alone. [5] In the absence of permanent end-organ damage, most drug overdoses follow a self-limiting course, with recovery occurring as the toxin is cleared via renal, hepatic, or extracorporeal elimination. [5], [29] Even though VA-ECMO is seen as an resuscitative measure, the use of VA-ECMO in the poisoned patient is still limited by availability, logistics of transport, patient comorbidities, and risks inherent to the procedure. [5].

Additionally, a 2023 systematic review [29] on ECMO in intoxicated patients highlighted some limitations: most published cases involved predominantly young patients (mean age 30.9 ± 16.6 years), and mainly single or double-agent intoxications rather than complex poly-intoxications with multiple interactions. This focus on younger patients limits the generalizability of the findings, as their greater physiological reserve likely contributes to better survival and tolerance of both intoxication and ECMO-related complications, rendering outcomes in older patients less certain. As a result, outcomes reported in such cohorts may be overly optimistic and not representative of older patients with more comorbidities. On the other hand, the paucity of data and selective reporting on younger patient might create a higher threshold to initiate VA-ECMO in older intoxicated patients, while there is currently no evidence to back up the magnitude of the assumed poorer prognosis in this group. Furthermore, poly-intoxications may differ meaningfully from single-agent overdoses, as multiple mechanisms of toxicity act simultaneously, which may result in more refractory shock, less predictable responses to antidotes, and greater physiological instability prior to ECMO initiation. From a physiologic perspective it stands to reason that VA-ECMO is predominantly helpful in supporting circulation in cardiogenic shock and of less additional value in profound vasodilation. Consequently, caution is warranted when extrapolating existing VA-ECMO outcomes to older patients or patients with complex poly-intoxications, underscoring the need for more representative data to better inform patient selection and timing of VA-ECMO in this population.

As a result of these limitations, the AHA emphasises a key knowledge gap: determining which poisoned patients genuinely benefit from VA-ECMO compared with the conventional critical care population. [5] This case adds to the limited body of evidence by demonstrating the feasibility of VA-ECMO support in an elderly patient with severe poly-intoxication and relevant comorbidity. VA-ECMO enabled survival of the acute toxic phase and provided time for clearance of the offending agents in the setting of otherwise refractory cardiogenic shock. However, the subsequent clinical course was prolonged and complicated by vascular complications, prolonged mechanical ventilation, ICU-acquired weakness, and ultimately progressive functional decline leading to death approximately eight months after the intoxication.

This case therefore illustrates both the potential and the caveats of VA-ECMO in elderly intoxicated patients. Although acute recovery from the toxic insult was achieved without irreversible neurological injury, survival was associated with substantial long-term morbidity and loss of functional independence. In elderly patients with severe intoxication, short-term physiological rescue should therefore not automatically be equated with favorable long-term outcome. Careful multidisciplinary evaluation remains essential, balancing the reversibility of the intoxication against baseline frailty, expected quality of life, anticipated ICU burden, and the likelihood of meaningful long-term recovery.

Furthermore, the patient continued to deteriorate despite extensive conventional treatment, while marked hemodynamic improvement and lactate clearance were observed following VA-ECMO initiation, suggesting that VA-ECMO contributed substantially to stabilization and recovery. Nevertheless, given the multimodal treatment approach, this effect cannot be unequivocally attributed to VA-ECMO alone.

## 4 Conclusion

This case illustrates that in severe toxic cardiogenic shock due to complex poly-intoxication, VA-ECMO can be a life-saving therapeutic option when conventional pharmacological and supportive measures fail to achieve hemodynamic stabilization. While advanced age and comorbidity warrant particular caution, selected elderly patients with potentially reversible toxic cardiogenic shock may still be candidates for VA-ECMO after careful multidisciplinary evaluation. At the same time, this case highlights that successful short-term resuscitation may come at the cost of substantial long-term morbidity and prolonged rehabilitation, emphasizing the need to consider both immediate survival and anticipated long-term outcome when evaluating VA-ECMO candidacy in elderly poisoned patients. Careful patient selection, early optimization of medical therapy, and multidisciplinary decision-making remain essential. Further data are needed to better define which patients with severe intoxication derive meaningful benefit from VA-ECMO and to guide timing and selection in this population.

## CRediT authorship contribution statement

**N. Kraaijvanger:** Writing – review & editing, Visualization, Supervision, Investigation, Conceptualization. **J.J.J. Ouwerkerk:** Writing – review & editing, Writing – original draft, Visualization, Software, Resources, Project administration, Investigation, Data curation, Conceptualization. **A.F. Westra:** Writing – review & editing, Visualization, Supervision, Investigation, Conceptualization. **N.A. Poublon:** Writing – review & editing, Visualization, Supervision, Investigation, Conceptualization.

## Declaration of Competing Interest

The authors declare that they have no known competing financial interests or personal relationships that could have appeared to influence the work reported in this paper.

## Data Availability

No data was used for the research described in the article.
